# Are Organizational Interventions Effective in Protecting Healthcare Worker Mental Health during Epidemics/Pandemics? A Systematic Literature Review

**DOI:** 10.3390/ijerph19159653

**Published:** 2022-08-05

**Authors:** Nektaria Nicolakakis, Maude Lafantaisie, Marie-Claude Letellier, Caroline Biron, Michel Vézina, Nathalie Jauvin, Maryline Vivion, Mariève Pelletier

**Affiliations:** 1 Department of Environmental and Occupational Health and Toxicology, Institut National de Santé Publique du Québec (INSPQ), Montreal, QC H2P 1E2, Canada; 2 Department of Public Health Gaspésie-Îles-de-la-Madeleine, Integrated Health and Social Services Centre of Gaspésie, Gaspe, QC G4X 1A9, Canada; 3 Department of Management, Laval University, Quebec City, QC G1V 0A6, Canada; 4 Department of Social and Preventive Medicine, Laval University, Quebec City, QC G1V 0A6, Canada; 5 Department of Scientific Valorisation and Quality, INSPQ, Montreal, QC H2P 1E2, Canada

**Keywords:** effectiveness evaluation, health and social services worker, infectious disease, pandemic, psychological health, psychosocial work environment, occupational determinants of stress

## Abstract

It is unclear how to effectively protect healthcare workers’ mental health during infectious disease epidemics. Targeting the occupational determinants of stress may hold more promise than individual stress management, which has received more focus. Through a systematic review of the 2000–2021 English- and French-language scientific literature, we evaluated the effectiveness of organizational and psychosocial work environment interventions to protect healthcare workers’ mental health in an epidemic/pandemic context. Evidence from medium- and high-quality studies was synthesized using GRADE. Among 1604 unique search results, 41 studies were deemed relevant, yielding 34 low-quality and seven medium-quality studies. The latter reported on promising multi-component prevention programs that combined staffing adjustments, work shift arrangements, enhanced infection prevention and control, recognition of workers’ efforts, psychological and/or logistic support during lockdowns (e.g., accommodation). Our confidence in the effectiveness of reviewed interventions is low to very low, however, owing to methodological limitations. We highlight gaps in the reporting of intervention process and context elements and discuss theory and implementation failure as possible explanations for results. We conclude by urging authors of future studies to include and document detailed risk assessments of the work environment, involve workers in solution design and implementation and consider how this process can be adapted during an emergency.

## 1. Introduction

The healthcare workforce experiences a substantial burden of ill mental health, burnout and turnover [1,2,3,4,5] and an increased burden of mental health symptoms and problems during epidemic and pandemic health emergencies, such as the COVID-19 pandemic [6,7]. The exacerbation of already suboptimal working conditions, including high demands and inadequate staffing [1,3,5,8], compounded by specific workplace stressors associated with epidemics or pandemics, seem to contribute to this burden. These stressors include lack of personal protective equipment (PPE), involuntary deployment, reassignment to unfamiliar teams and tasks, increased work-family conflict related to school and daycare closures and the experience of moral dilemmas when caring for infected patients while risking one’s own health and that of one’s family and when having to decrease the quality of care due to resource constraints [6,7,8,9,10,11,12,13].

It has been suggested that 44% of hospital turnover could be prevented through hypothetical improvements in the psychosocial work environment [14]. In their meta-analysis, Panagioti and colleagues [15] showed that organization-directed compared to person-directed interventions were more effective in protecting physicians against burnout. Evidence on the effectiveness of work environment interventions in protecting healthcare workers’ mental health in an epidemic or pandemic context is limited. As Muller et al. [16] stated in their COVID-19 rapid review, there is “a mismatch between the likely organizational sources of psychological distress […] and how healthcare systems are attempting to relieve distress at an individual level.” A mixed-methods review on interventions (not limited to organizational interventions) covering the period 2002 to 28 May 2020, identified only one study reporting on the effectiveness of workplace-delivered psychological first aid training to frontline healthcare staff to assist the Sierra Leone population after the Ebola outbreak (non-randomized studies were excluded from that review) [17]. Soklaridis et al. [18], covering studies on mental health interventions during epidemics published between 2003 and 31 July 2020, reported mainly on individual-level behavioral interventions (e.g., cognitive behavioral therapy (CBT), music therapy] and on a single organizational intervention among health professionals that has been captured in the present review [19]. The systematic review by Zace et al. [20] covering mental health intervention studies published up to 2 October 2020 included studies that either did not report on effectiveness with respect to a mental health outcome and/or were not directed at the work environment. It is also useful to search for relevant literature published since the aforementioned reviews.

Our goal was to conduct a systematic review of the scientific literature in order to answer the following research question: compared to usual work (i.e., no intervention), what is the effectiveness of organizational and psychosocial work environment interventions in protecting healthcare workers’ mental health in an infectious disease epidemic/pandemic context? By work organization, we refer to the way in which work is designed and performed, including the nature and distribution of work tasks, production methods, work pace, management, scheduling, remuneration, and training practices and policies [21]. The psychosocial work environment results from the interplay between the working conditions, work organization, management practices and social relations at work. It refers to, among others, the intensity of physical, emotional and cognitive work demands (e.g., excessive workload, time constraints), the level of control over one’s own work, the possibility for skill development and creativity at work, emotional and practical support from supervisors and colleagues, recognition of worker efforts (e.g., in the form of job security, respect, promotion prospects and salary), work-life balance and workplace harassment and violence [22,23,24,25,26,27,28,29]. In this review, we include workplace interventions targeting work organization or any of the aforementioned elements of the psychosocial work environment in order to protect mental health. We also include organizational interventions targeting the epidemic-causing biological hazard to protect mental health, for example through infection prevention and control (IPC) protocols and training. The fear of infection may be a risk factor for mental health problems in healthcare personnel during epidemics [6] and mitigating the biological risk could therefore be protective. Moreover, men and women may have different physical and psychosocial work exposures, thus different intervention needs (e.g., daycare closures during lockdowns may impact work-family balance differently for female compared to male healthcare staff). We therefore also examined whether such considerations were present in the analyzed studies, either in the study theoretical framework (e.g., introduction) or during data collection, analysis or interpretation.

## 2. Materials and Methods

This review is reported according to the Preferred Reporting Items for Systematic Reviews and Meta-Analyses (PRISMA) statement [30]. The review was not registered. The review protocol (in French) can be obtained upon request from the authors.

### 2.1. Literature Search

We searched for English- and French-language peer-reviewed scientific studies published between 1 January 2000, and 9 September 2021, in nine electronic databases: Medline, Excerpta Medica Database (Embase), EBM reviews/Cochrane Library, Cumulative Index to Nursing and Allied Health Literature (CINAHL), PsycINFO, SocINDEX, Psychology and Behavioral Sciences Collection, Business Source Premier and Social Science Abstracts (access to the latter two databases was available to us until 22 December 2020). In addition, we manually searched the reference lists of eligible studies and reviews. The search strategy was developed with the help of a librarian and combined natural language and database-specific terms using Boolean logic and proximity operators. Terms referred to four broad concepts: (1) mental health problems, (2) organizational or psychosocial work environment interventions, (3) effectiveness evaluation and (4) epidemic/pandemic. A detailed search strategy for Medline is provided in Appendix A, Table A1.

### 2.2. Study Eligibility and Exclusion

Eligible studies were epidemiologic studies reporting on an organizational or psychosocial work environment intervention to protect the mental health of healthcare workers (including managers, team leaders, and heads of health and social services establishments) in an infectious disease epidemic or pandemic context (e.g., COVID-19, severe acute respiratory syndrome (SARS), middle east respiratory syndrome (MERS), influenza, H1N1 flu, Zika virus disease, Ebola virus disease), that reported on the effectiveness of the intervention on a mental health outcome or psychosocial work environment indicator. Most often, the effects of the solutions or measures generated by the intervention process are reported and quantified in epidemiological evaluation studies. We therefore also included studies that reported on associations between preventive measures and mental health or work exposure indicators, even if an intervention *per se* had not been described. We penalized such studies during methodologic quality assessment for insufficient information on contextual and implementation elements. Moreover, given the challenges associated with conducting and evaluating complex system-level interventions in dynamic work settings and the anticipated paucity of randomized trials in work environment research [31,32], especially in an epidemic context, we also included observational study designs, though these were penalized during quality assessment. We excluded studies focusing solely on individual-level stress management interventions that did not make changes to the work environment (e.g., CBT, mindfulness training, fitness programs).

Search results were exported to Zotero, and duplicates were removed. Two authors independently screened records against the selection criteria, first by reading the title and abstract, then the full text of records initially retained. Disagreements were resolved through discussion to reach consensus, and when necessary, through decision by a third author.

### 2.3. Methodologic Quality Assessment of Individual Studies

We evaluated the methodologic quality of selected studies with the 15-item instrument used by Stock et al. [33] and adapted to the current study. The instrument addresses various sources of bias related to study design, selection, attrition, measurement and confounding, and includes workplace intervention-specific items pertaining to implementation, co-interventions and contextual factors (Appendix A, Table A2). Scores range from 0 to 2 points at the item level for a maximum study score of 30 points. Total study scores were converted to percentages and study quality was categorized as low (0–50%), medium (51–79%) or high (80–100%). To promote inter-rater reliability, item interpretation was tested on two studies and discussed prior to independent critical appraisal of the remaining studies by two authors. Consensus was sought at the item and study level. Only studies of at least medium quality were retained for data extraction and analysis.

### 2.4. Data Extraction and Analysis

The following information was extracted by one author from studies of sufficient quality, and confirmed by a second author: country of research, study design, sample size, participant characteristics [sex (biological attribute) and/or gender (encompasses socio-culturally-shaped attitudes, behaviours and roles), occupation], participation and drop-out rate, intervention content, implementation, duration and timing of follow-up, mental health indicators, indicators of exposure to the epidemic-causing pathogen and to elements of the psychosocial work environment, confounding variables, co-interventions and contextual factors considered, statistical analysis and effect of the intervention on the mental health and/or work exposure indicators (e.g., the difference in prevalence or in mean score). We contacted the authors of three studies for clarifications (Supplementary Table S1). It was not possible to pool results for meta-analysis due to heterogeneous interventions (in content and duration) and outcomes across the few studies of sufficient quality that were retained for analysis.

### 2.5. Evidence Synthesis

For each outcome measure reported in the studies for a given intervention, the quality of the evidence on effectiveness was assessed as high, moderate, low or very low, reflecting our level of confidence in effect estimates, according to the Grading of Recommendations Assessment, Development and Evaluation (GRADE) approach. Randomized trials provide initially high-quality evidence in GRADE that can be rated down by one or two levels if there is a risk or serious risk of bias (methodologic limitations), inconsistency, indirectness, imprecision or publication bias. Observational studies provide initially low-quality evidence, but in the absence of methodologic limitations, can be rated up if there are large effects across studies, for example [34,35,36,37,38,39,40,41].

## 3. Results

### 3.1. Identification and Selection of Studies

**Figure 1:**
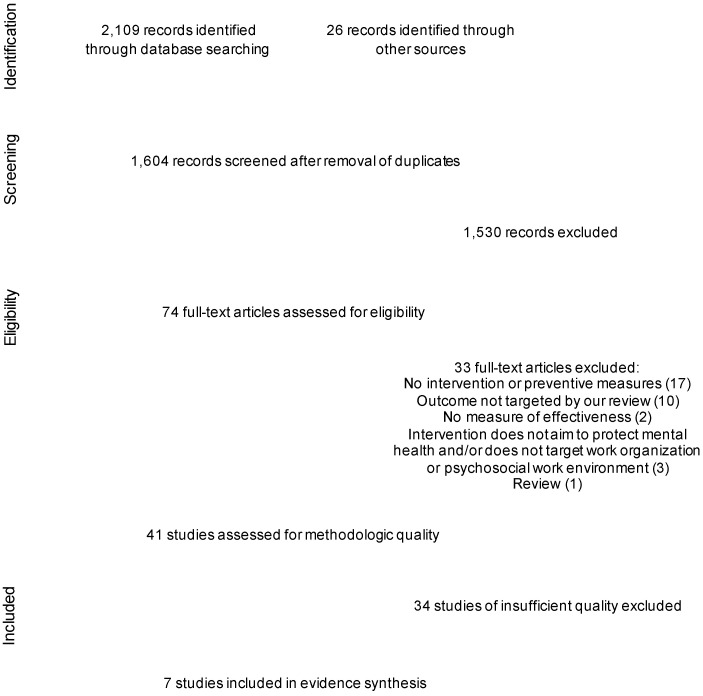
PRISMA flowchart detailing the identification and selection of studies.

Figure 1 presents the number of identified and selected studies. The search produced 1604 unique records, of which 1530 were excluded based on their title and abstract, and 74 retained for further assessment of their full text. Of these, we excluded 33 not meeting selection criteria, leaving 41 studies that were assessed for methodologic quality.

### 3.2. Methodologic Quality of Individual Studies

The methodologic quality assessment gave rise to 34 low-quality and seven medium-quality studies (Table 1). Overall, studies performed poorly on item 2 (lack of a control group), items 4 and 5 (relevant work exposures not measured and inappropriate indicators), item 9 (undocumented or low participation rate at recruitment), items 10 and 11 (undocumented or important loss to follow-up without a comparison of study “completers” and “drop-outs”; these two items were also used to penalize for cross-sectional designs), item 12 (undocumented implementation of targeted changes or none/few changes implemented) and item 14 (co-interventions and contextual changes not documented or few were documented or considered in analysis or result interpretation).

### 3.3. Description of Analyzed Studies and Interventions

A description of the seven studies retained for data extraction and analysis is presented in Supplementary Table S1. Five studies were conducted during the COVID-19 pandemic [42,43,44,45,46], two in the context of SARS [19,47]. Studies were from Canada [47], the United Kingdom [43], Italy [44,45], Spain [42], China [46] and Taiwan [19]. Study designs included a controlled prospective cohort study [42], three before-after uncontrolled studies [19,44,45] and three cross-sectional studies [43,46,47]. Samples were predominantly female (at least 85% in five studies) and included mainly nursing professionals (>65% in four studies) in hospital settings. The seven studies collected information on sex or gender through online questionnaire, offering the categories male and female (though these were not defined), and one study reported an additional non-binary/gender fluid category [43]. One or more mental health outcomes were measured in the studies, including but not limited to anxiety, depression and post-traumatic stress, and related outcomes, such as sleep quality, intention to quit and presenteeism.

**Table 1:** Methodologic quality of individual studies.

Studies	Items ^1^															Study Score		Study Quality ^2^
	1	2	3	4	5	6	7	8	9	10	11	12	13	14	15	/30	%	
Zaghini et al. 2021 [45]	2	0	2	2	1.5	2	1.5	2	2	2	2	1.5	1	0	1	22.5	75.0	M
Giordano et al. 2021 [44]	2	0	1.5	1.5	1.5	2	1	2	0	2	2	2	0.5	0	1	19.0	63.3	M
Chen et al. 2006 [19]	2	0	1.5	1	0	2	1.5	2	0	2	2	1	1.5	0	1	17.5	58.3	M
Maunder et al. 2006 [47]	2	1	2	1	1	1	2	2	1.5	0	0	0.5	1.5	0	2	17.5	58.3	M
Zhu et al. 2020 [46]	2	1	1.5	1	0.5	1	1.5	1	2	0	0	1.5	2	0.5	1.5	17.0	56.7	M
Beneria et al. 2020 [42]	2	1	1	1	1	1	1	2	1	0.5	0	2	1	0.5	0.5	15.5	51.7	M
Blake et al. 2020 [43]	2	1	1	1	0.5	1	1.5	2	0.5	0	0	2	1	1	1	15.5	51.7	M
Chen et al. 2021	2	0	1.5	1	1.5	1	1.5	0.5	0.5	0	0	0.5	2	1	2	15.0	50.0	L
Cyr et al. 2021	2	1	2	1	1	1	1.5	1	0	0	0	0.5	2	0.5	1.5	15.0	50.0	L
Smith et al. 2020	2	1	1.5	1	1	1	1.5	0.5	0.5	0	0	0.5	1.5	1	2	15.0	50.0	L
Arnetz et al. 2020	2	1	1	1	1	1	2	1	0.5	0	0	0.5	1.5	0	2	14.5	48.3	L
Lancee et al. 2008	2	0	1	1	1	1	2	2	1.5	0	0	0.5	1	0	1.5	14.5	48.3	L
Xu et al. 2021	2	0	1	0.5	0.5	2	1.5	1	1	1	0	2	0	0	1.5	14.0	46.7	L
Tam et al. 2004	2	1	1.5	1	0.5	1	1.5	2	0	0	0	0.5	1	0	2	14.0	46.7	L
Lasalvia et al. 2021	2	1	2	1	1	1	1.5	1.5	0	0	0	0.5	1	0	1	13.5	45.0	L
Castro-Sanchez et al. 2020	2	1	1	1	0.5	1	0	2	0	0	0	2	1	0.5	1.5	13.5	45.0	L
Zhan et al. 2020	2	1	1.5	1	1	1	1.5	0	0	0	0	0	1.5	1.5	1.5	13.5	45.0	L
Hennein et al. 2021	2	0	2	1	1	1	1.5	1	0	0	0	0.5	2	0	1	13.0	43.3	L
Sharma et al. 2021	2	1	1.5	1	1	2	2	0	0	0	0	0.5	1	0	1	13.0	43.3	L
Huang et al. 2020	2	1	1	1	1	1	1.5	1	0	0	0	0.5	2	0	1	13.0	43.3	L
Chan and Huak 2004	2	0	0.5	0.5	1	1	1.5	1.5	1	0	0	0	1	1	2	13.0	43.3	L
Matsuishi et al. 2012	2	0	1.5	1	0.5	1	1.5	2	0	0	0	0.5	1	0	2	13.0	43.3	L
Fiksenbaum et al. 2006	2	0	1	1	1	1	2	2	0	0	0	0	0.5	0	2	12.5	41.7	L
Marjanovic et al. 2007	2	0	1	1	1	1	2	2	0	0	0	0	0.5	0	2	12.5	41.7	L
Petrella et al. 2021	2	1	1	0	0	1	1.5	1	0	0	0	1.5	1.5	0	1.5	12.0	40.0	L
Esmaeilzadeh et al. 2021	2	0	1.5	1	1	1	1	1	1	0	0	0.5	1	0.5	0.5	12.0	40.0	L
Holton et al. 2020	2	0	1	1	0.5	1	2	0.5	0.5	0	0	0	1	1	1.5	12.0	40.0	L
Kim and Choi 2016	2	0	1.5	1	0.5	1	1.5	1.5	0	0	0	0	1	0	2	12.0	40.0	L
Kase et al. 2021	2	1	1	1	1	1	1.5	0	0	0	0	0.5	1.5	0	1	11.5	38.3	L
Young et al. 2021	2	1	1	0	0	1	1.5	1	0	0	0	0.5	1.5	0	2	11.5	38.3	L
Morgantini et al. 2020	2	1	1	1	1	1	1	0.5	0	0	0	0.5	1	0	1.5	11.5	38.3	L
Demirjian et al. 2020	2	1	1.5	1	0.5	1	1	1	0	0	0	0	1	0	1.5	11.5	38.3	L
Durmaz Engin et al. 2021	2	0	1	1	1	1	1.5	1	0	0	0	0.5	1	0	1	11.0	36.7	L
Buch et al. 2021	2	0	1	1	0	1	1	2	0.5	0	0	1.5	0	0	0.5	10.5	35.0	L
Shalhub et al. 2020	2	0	1	1	1	1	1.5	0	0	0	0	0.5	0.5	0	1.5	10.0	33.3	L
Martinez-Caballero et al. 2021	2	0	1	0	0	1	1.5	1	0	0	0	1	1	0	1	9.5	31.7	L
Temsah et al. 2021	2	1	0.5	0	0	1	1	0	0	0	0	0.5	1.5	0	2	9.5	31.7	L
Zhang et al. 2020	1.5	0	1.5	1	0	1	1.5	1	0	0	0	0	1	0	1	9.5	31.7	L
Cai et al. 2020	2	0	0.5	0.5	0	1	0	1	1	0	0	1	0	0.5	0.5	8.0	26.7	L
Huffman et al. 2020	2	0	0.5	1	0.5	1	0	0.5	0	0	0	0	1	1	0	7.5	25.0	L
Reidy et al. 2020	2	0	0.5	1	0	1	0	1	0	0	0	1	0.5	0	0.5	7.5	25.0	L

^1^ The 15 quality assessment items refer to those presented in Table A2. ^2^ M: medium; L: low. The complete references for low-quality studies are available upon request to the authors.

Interventions were described in six studies. Authors of the seventh study [47] reported the association between perceived adequacy of training, protection and support measures and mental health outcomes, but did not describe an intervention *per se*. Intervention content and duration varied. Blake et al. [43] described the implementation and usage of wellbeing centers over approximately four months. The centers consisted of one purpose-built room and one converted hospital ward equipped with staff (‘wellbeing buddies’) trained to offer psychological first aid to personnel of an acute hospital trust (listening, comforting and directing towards services, as needed). Wellbeing buddies were employees with a reduced workload because of the pandemic, who volunteered for the role and received training and supervision by two clinical psychologists. Beneria et al. [42] described a 25-h simulation-based teamwork training program aimed at developing leadership and communication skills required in a crisis. The study by Giordano et al. [44] reported on the “R2 for Leaders” resilience training program consisting of 12 virtual two-hour weekly sessions over three months. It was intended to equip healthcare leaders to better lead their staff and their organization through the identification and implementation of individual-level as well as organizational prevention programs (details in Supplementary Table S1).

Three studies described multi-component programs combining slightly different preventive measures, lasting approximately two weeks [46], three months [19] and four months [45]. The two-week program reported by Zhu et al. [46] was initiated by hospital management at Wuhan’s largest tertiary hospital designated for the treatment of severe COVID-19 patients. It included several measures targeting workplace recognition, such as additional allowances for frontline staff, verbal recognition and reassurance by hospital executives, nursing leaders and department chairs, and acknowledging staff’s infections as work injuries. Measures to protect against nosocomial infection included the use of PPE in all departments, regardless of the presence of infected patients. The program also included what authors referred to as “reasonable” work shift arrangements, workplace meals and hydration, and the arrangement by hospital administrators of shuttle services, hotel rooms and dormitories when public transport was suspended by authorities, to reduce the staff’s fear of infecting their family. A virtual support group led by the hospital psychiatry team was also organized, though used by only 5% of the staff, perhaps due to workers’ concerns over confidentiality or stigmatization, as hypothesized by the authors.

In the study by Zaghini et al. [45], an Italian university hospital proactively started to prepare for the arrival of the pandemic in order to manage its impacts on the nursing staff. The hospital reorganized its wards (e.g., increasing intensive care beds), procedures (e.g., cleaning and disinfection) and internal paths to separate infected from uninfected patients. Nurse-to-patient ratios were increased, from 1:9 to 1:6 in COVID units of medium-intensity care and from 1:4 to 1:2 in high-intensity care units, maintaining these ratios over 24 h. Nurses were provided with training on the correct use of PPE and were monitored for infection through COVID-19 testing. A psychological help desk was established, available to staff every day on-site and remotely. The hospital promoted a participatory approach and autonomy, for example, through meetings where nurses could discuss the care of critical cases with other healthcare professionals. In focus groups, nurses expressed a greater sense of autonomy, with statements such as “doctors and managers had never asked us our opinion on how to perform a certain intervention on a patient, but in the SARS-CoV-2 context, they did!” and “suddenly we were autonomous professionals in a process that was unfamiliar to everyone; they asked us for opinions and gave us the opportunity to experiment with solutions that we found independently”.

The three-month program described by Chen and colleagues [19] was initiated by a SARS-designated treatment hospital in Taiwan. It comprised limiting the workday to eight hours to prevent fatigue, adjusting staffing levels according to the number of admitted SARS patients, alternating the units that treated SARS patients on a weekly basis, daily information updates to workers, availability of immune-boosting supplements to nursing staff, availability of PPE, a variety of IPC measures, protocols and in-service training (53 classes) for the handling of SARS patients and the correct use of PPE, and the availability of a multidisciplinary mental health team and clinic for workers.

The effects of the interventions were analyzed in combined samples of men and women in the seven studies. Three of the studies adjusted regression analyses for the sex variable [42,46,47]. One study additionally carried out stratified analyses in men and women separately [46], yielding slightly different intervention effects (details in Supplementary Table S1). Namely, in women, most measures seemed protective (recognition measures, satisfaction with reasonable work shift arrangements and with logistic support, i.e., workplace-provided meals, transportation and accommodations), whereas the only factor that appeared to be protective in men was satisfaction with IPC measures. These findings were reported as supplementary material, but not addressed in the main paper, besides the brief mention in the discussion of “entrenched traditional social roles in China” leading to dilemmas for women “between working and family care and between the family care and avoidance of contact with family members” [46]. Stratified analyses were not possible for most studies because of the small number of men in the samples. Sex and gender considerations were absent in the theoretical framework of the studies and absent [19,43,44,45,47] or minimal [42,46] in result interpretation.

### 3.4. Quality of the Evidence on Intervention Effectiveness

Table 2 summarizes the quality of the evidence on intervention effectiveness for each outcome measure reported in the studies. The aforementioned multi-component prevention programs appear to be protective, reducing, for example, the likelihood or level of anxiety and depression [19,46] or improving the quality of the psychosocial work environment or of some of its dimensions like job control, managerial and peer support and the quality of relationships at work [45]. However, our confidence in the effectiveness of these and other reviewed interventions is low to very low owing to the observational study designs and serious risks of selection and confounding bias. Notably, most studies failed to describe the intervention process and implementation as well as context elements that may influence intervention effectiveness [48] (Table 2 and Supplementary Table S1).

## 4. Discussion

Based on a rather small number of studies, we found low- to very low-quality evidence on the effectiveness or ineffectiveness of the reviewed organizational or psychosocial work environment interventions on healthcare workers’ mental health during an epidemic or pandemic context. Our level of confidence in effect estimates is therefore low to very low, and real intervention effects are likely very different from those estimated in the analyzed studies [41]. Nevertheless, promising solutions were evaluated in these studies that may warrant consideration in future research.

### 4.1. Theory or Implementation Failure?

An important question is whether the interventions reviewed in the current study are theoretically likely to protect mental health [31]. More specifically, were they designed to target the work exposures contributing to stress and psychological ill health and did they mitigate these harmful exposures? These questions remain largely unanswered, as intervention effects on working conditions and on indicators of the psychosocial work environment were often undocumented. Some of the barriers to the use of wellbeing centers [43] included that it was not possible to take a break or that breaks were too short. Although access to a space for respite and psychological support could be useful in the context of a broader prevention program, could work–rest schedules or staffing ratios [49] have been the necessary targets for intervention to reduce the prevalence of presenteeism and intention to quit? Staffing issues were alluded to as a potential reason for missed breaks by authors of the wellbeing centers study [43]. In Beneria et al. [42], simulation-based teamwork training failed to mitigate the likelihood of anxiety and depression. It is unclear if this is partly due to a failure to improve workplace communication, teamwork and leadership targeted by the intervention after a single 25-h course, or if the intervention missed the predominant causes of occupational stress.

Secondly, assuming that interventions correctly diagnosed the occupational determinants of stress, were interventions implemented as intended and were changes integrated by workers into their practices? Such details were rarely provided in the studies, and when they were, information was limited. For example, in the study evaluating a three-month SARS prevention program, no information was given regarding availability of PPE across units, adherence to IPC protocols, worker awareness and participation in training and awareness and use of the mental health clinic [19]. Regarding the COVID-19 prevention program reported by Zhu et al. [46], details were not provided on whether PPE availability varied across hospital departments, whether “reasonable” work shifts were negotiated or set by management, whether shuttle services were readily or intermittently available and whether the staff was aware that hospital-acquired infection could be recognized as a work injury and what this recognition process entailed (e.g., a complicated process could have had unintended negative effects). Such details could have shed light on divergent results between men and women reported by the authors in stratified analyses. They would help to determine if, for example, certain components of the program were integrated differently by male and female staff. This also highlights more generally the importance of conducting distinct analyses in men and women, where sample size permits, and the study by Zhu et al. [46] was one of the few to do so, of all studies we reviewed. Adjustment for the sex/gender variable, rather than stratification, was the norm in reviewed studies, a practice that may have concealed distinct associations in men and women [50,51,52]. Considering that men and women often vary in their personal [53] and professional exposures [54], as well as in their interactions with health and compensation systems [55,56,57], research will be enhanced by considering exposure-outcome relations and intervention effects in the male and female workforce [58]. This type of subgroup analysis is consistent with the recommendations for realist evaluation to determine for whom, when and in what context interventions produce intended effects [59].

Information on other initiatives (co-interventions) occurring alongside the main intervention was similarly lacking in most studies, as was information on workplace dynamics and contextual changes that may have affected how preventive measures were applied and followed (e.g., labor disputes, staff turnover, changes in management, rapidly changing public health guidelines). Moreover, in nearly all studies reviewed, workplace actors appear not to have been involved in risk assessment or solution development or if they were, this was not documented. Workers are uniquely positioned to identify risks to their health and contribute to solutions that are compatible with their work and their wellbeing. Participatory intervention processes that include employees and managers at different levels within the organization can facilitate the implementation of changes and increase their uptake, thereby enhancing intervention effectiveness [60,61]. Several authors have argued for the involvement of key stakeholders and end-users in intervention design and implementation and for the consideration of context and process elements when evaluating outcome effects [48,60,62,63,64,65,66]. Innovative approaches for studying the mechanisms through which participatory organizational interventions exert their effects have been put forth and could help advance intervention evaluation research [67].

### 4.2. Strengths and Limitations

This is one of the few systematic reviews on the effectiveness of organizational and psychosocial work environment interventions to protect healthcare worker mental health in an epidemic or pandemic context. Outside of an infectious disease emergency context, such interventions are also relatively scarce [15,68]; a preponderance of mental health intervention studies have evaluated individual stress management like CBT. This study is therefore one of the relatively few to attempt to shift the focus from individual stress relief towards upstream workplace prevention targets that can produce broader and more durable effects. We used a systematic approach and author consensus in study identification, selection and evaluation, in an attempt to reduce bias. The search covered an extensive literature in English and French spanning 21 years across nine databases (and 20 years across 7 databases) in medicine, nursing, psychology, sociology and business. The sensitivity of the search strategy was tested, and the strategy was adapted with the help of a librarian. However, due to time constraints, non-peer-reviewed pre-publications and gray literature were not included and some intervention studies may have been missed. Some of our requests for clarifications to authors of original studies were not answered, therefore conclusions are based on an accurate interpretation of related findings.

## 5. Conclusions

We identified very few organizational or psychosocial work environment interventions to protect healthcare workers’ mental health during epidemics/pandemics, and these provided low- to very-low quality evidence on (in)effectiveness. There was also a gap in the reporting of intervention process and context elements that could account for outcome effects. Nevertheless, several promising solutions in the studies reviewed herein may help orient future efforts and ultimately contribute to building more robust healthcare systems that can withstand the challenges of new health emergencies. Authors of future intervention studies should consider carrying out and reporting detailed risk assessments of the work environment, a participatory approach that mobilizes key workplace stakeholders, context and process evaluation to allow for adequate interpretation of intervention effects [48,60,63,65,67], as well as distinct analyses in men and women, where sample size permits. Unique challenges associated with a health emergency will need to be considered and will likely require adapting the intervention process. For example, virtual stakeholder consultations rather than in-person focus group meetings may be needed that meet physical distancing requirements while giving a voice to workers. High-impact solutions that can be implemented rapidly may need to be prioritized during an emergency. Ensuring that interventions are theoretically designed to address the occupational determinants of stress and that workers are involved in change processes should increase the likelihood of better health outcomes for the healthcare workforce during the COVID-19 pandemic and beyond.

## Figures and Tables

**Table 2:** Summary of intervention effects on mental health or psychosocial work exposure indicators and quality of the evidence on intervention effectiveness according to the Grading of Recommendations Assessment, Development and Evaluation (GRADE) approach.

Intervention ^1^	Intervention Effect ^1^ on Mental Health or Psychosocial Work Exposure Indicators	Quality of the Evidence ^2^ on Intervention Effectiveness and Justification of Rating
Simulation-based teamwork training program (25 h) (Beneria et al. 2020) [42]	**↑ likelihood of anxiety and depression** (HADS > 12) post-program in workers having had contact with COVID-19 patients: AOR 2.56, 95% CI: 1.03–6.36; *p* = 0.043. AOR not reported for all workers who received the training program or for those who received it but had had no contact with COVID-19 patients.	**Very low** Observational design, serious risks of selection and confounding bias (48% participation rate for control group, important confounders omitted, i.e., history of mental illness, psychosocial work exposures)
Wellbeing centers supported by wellbeing buddies (4–5 months) (Blake et al. 2020) [43]	**↑ mental wellbeing** of 1.93 points on WEMWBS scale that ranges from 14 to 70 points: mean WEMWBS score (SD) is 47.04 (9.49) for center users and 45.11 (9.35) for non-users; *p* = 0.02	**Very low** Observational design, serious risks of selection and confounding bias (<5% participation rate, confounding factors not considered in analysis)
	**↑ work engagement** of 0.19 points on dedication subscale of UWES-9 ranging from 0 to 6 points: mean score (SD) is 5.02 (1.38) for center users and 4.83 (1.15) for non-users; *p* = 0.08	**Very low** Observational design, serious risks of selection and confounding bias (<5% participation rate, confounding factors not considered in analysis)
	**≠ % presenteeism past 12 months** among center users vs. non-users: no, never: 16.31 vs. 14.97 yes, once: 17.05 vs. 12.76 yes, 2 to 5 times: 16.92 vs. 12.64 yes, >5 times: 4.53 vs. 4.41 *p* = 0.28	**Very low** Observational design, serious risks of selection and confounding bias (<5% participation rate, confounding factors not considered in analysis)
	**≠ % with intention to quit** among center users (16.31%) and non-users (15.09%); *p* = 0.25	**Very low** Observational design, serious risks of selection and confounding bias (<5% participation rate, confounding factors not considered in analysis)
Multi-component SARS prevention program: scheduling and staffing adjustments, IPC measures and protocols, latest PPE, daily information, training, mental health team and clinic for workers (3 months) (Chen et al. 2006) [19]	**↓ anxiety level** from moderate before SARS patient care (T0) to mild two weeks (T1) and one month (T2) under prevention program to no anxiety at final time point (T3), Zung’s self-rating anxiety scale: Mean anxiety score (SD) T0: 60 (9.28) T1: 51 (10.32) T2: 50 (9.84) T3: 46 (7.48) Change (improvement) ○ T0 vs. T1: Z = −2.68; *p* = 0.0075 ○ T0 vs. T2: Z = −4.45; *p* < 0.0001 ○ T0 vs. T3: Z = −6.58; *p* < 0.0001	**Very low** Observational design, serious risks of selection and confounding bias (participation rate ND, unclear if all measured covariables were included in models, other potential confounders not measured, i.e., program compliance, medication use for anxiety, work exposures, e.g., changing work schedules mentioned in discussion, other factors outside work)
	**↓ depression level** from moderate before SARS patient care (T0) to mild two weeks (T1) and one month (T2) under prevention program to no depression at final time point (T3), Zung’s self-rating depression scale: Mean depression score (SD) T0: 61 (12.62) T1: 51 (11.94) T2: 50 (10.60) T3: 48 (10.76) Change (improvement) ○ T0 vs. T1: Z = −4.58; *p* < 0.0001 ○ T0 vs. T2: Z = −4.80; *p* < 0.0001 ○ T0 vs. T3: Z = −6.37; *p* < 0.0001	**Very low** Observational design, serious risks of selection and confounding bias (participation rate ND, unclear if all measured covariables were included in models, other potential confounders not measured, i.e., program compliance, medication use for depression, work exposures, e.g., changing work schedules mentioned in discussion, other factors outside work)
	**↑ sleep quality** under prevention program, but sleep quality remains poor, i.e., PSQI > 5, at all time points Mean sleep quality score (SD) T0: 12 (3.83) T1: 10 (3.43) T2: 10 (3.77) T3: 8 (2.75) Change (improvement) ○ T0 vs. T1: Z = −2.79; *p* = 0.0053 ○ T0 vs. T2: Z = −3.14; *p* = 0.0017 ○ T0 vs. T3: Z = −3.37; *p* = 0.0008	**Very low** Observational design, serious risks of selection and confounding bias (participation rate ND, unclear if all measured covariables were included in models, other potential confounders not measured, i.e., program compliance, work exposures, e.g., changing work schedules mentioned in discussion, other factors outside work)
Multi-component COVID-19 prevention program: recognition measures (2 weeks) (Zhu et al. 2020) [46]	Recognition measures are associated with **24% ↓ likelihood of anxiety** (GAD-7 ≥ 8) compared to not having received recognition measures: AOR (95% CI): 0.76 (0.60–0.97); *p* = 0.03	**Low** Observational design
	Recognition measures are associated with **31% ↓ likelihood of depression** (PHQ-9 ≥ 10) compared to not having received recognition measures: AOR (95% CI): 0.69 (0.52–0.90); *p* = 0.007	**Low** Observational design
	Recognition measures are associated with **24% ↓ likelihood of acute stress** in the past 7 days caused by a traumatic event, COVID-19 being the specific event (IES-R > 33), compared to not having received recognition measures: AOR (95% CI): 0.76 (0.60–0.97); *p* = 0.024	**Low** Observational design
Multi-component COVID-19 prevention program: satisfaction with IPC measures (2 weeks) (Zhu et al. 2020) [46]	Satisfaction with IPC measures is associated with **35% ↓ likelihood of anxiety** (GAD-7 ≥ 8) compared to not being satisfied: AOR (95% CI): 0.65 (0.50–0.85); *p* = 0.002	**Low** Observational design
	Satisfaction with IPC measures is associated with **30% ↓ likelihood of depression** (PHQ-9 ≥ 10) compared to not being satisfied: AOR (95% CI): 0.70 (0.51–0.95); *p* = 0.02	**Low** Observational design
	Satisfaction with IPC measures is associated with **31% ↓ likelihood of acute stress** in the past 7 days caused by a traumatic event, COVID-19 being the specific event (IES-R > 33) compared to not being satisfied: AOR (95% CI): 0.69 (0.53–0.89); *p* = 0.004	**Low** Observational design
Multi-component COVID-19 prevention program: satisfaction with logistic support (shuttle service, meals/hydration and accommodation) (2 weeks) (Zhu et al. 2020) [46]	Satisfaction with logistic support is associated with **31% ↓ likelihood of anxiety** (GAD-7 ≥ 8) compared to not being satisfied: AOR (95% CI): 0.69 (0.50–0.96); *p* = 0.03	**Low** Observational design
	Satisfaction with logistic support is associated with **33% ↓ likelihood of depression** (PHQ-9 ≥ 10) compared to not being satisfied: AOR (95% CI): 0.67 (0.47–0.97); *p* = 0.03	**Low** Observational design
	Effect of satisfaction with logistic support on likelihood of acute stress in the past 7 days caused by a traumatic event, COVID-19 being the specific event (IES-R > 33), **not reported** because not significant in univariate analysis	**Low** Observational design
Multi-component COVID-19 prevention program: satisfaction with work shift arrangements (2 weeks) (Zhu et al. 2020) [46]	Effect of satisfaction with work shift arrangements on likelihood of anxiety **not reported** because not significant in univariate analysis	**Low** Observational design
	Satisfaction with work shift arrangements is associated with **52% ↓ likelihood of depression** (PHQ-9 ≥ 10) compared to not being satisfied: AOR (95% CI): 0.48 (0.34–0.67); *p* < 0.001	**Low** Observational design
	Satisfaction with work shift arrangements is associated with **55% ↓ likelihood of acute stress** in the past 7 days caused by a traumatic event, COVID-19 being the specific event (IES-R > 33), compared to not being satisfied: AOR (95% CI): 0.45 (0.33–0.63); *p* < 0.001	**Low** Observational design
“R2 for Leaders” resilience training program intended to equip healthcare leaders to better lead their staff and organization by identifying and implementing individual resilience and organization-level prevention programs (12 virtual 2-h weekly sessions over 3 months) (Giordano et al. 2021) [44]	**↓ emotional exhaustion level in healthcare leaders** post-program: mean MBI-EE score (SD): T1: 6.31 (1.35) vs. T2: 5.37 (1.20); *p* = 0.020; Hedge’s g (corrected Cohen’s *d* for small samples < 50) = −0.30	**Very low** Observational design, serious risks of selection and confounding bias (participation rate ND, potential confounders not considered in analyses), potentially inadequate power
	**≠ emotional exhaustion level in staff** post-program (no clinically or statistically significant difference): mean MBI-EE score (SD): T1: 4.70 (1.63) vs. T2: 4.35 (1.64); *p* = 0.098	**Very low** Observational design, serious risks of selection and confounding bias (participation rate ND, potential confounders not considered in analyses), potentially inadequate power
	**≠ quality of leaders’ psychosocial work environment** post-program: mean HSE-MSIT score (SD) on scale of 22 to 110: T1: 50.50 (15.33) vs. T2: 50.56 (15.17); *p* = 0.966	**Very low** Observational design, serious risks of selection and confounding bias (participation rate ND, potential confounders not considered in analyses), potentially inadequate power
	**↑ quality of staff’s psychosocial work environment** post-program: mean HSE-MSIT score (SD) on scale of 22 to 110: T1: 50.18 (10.56) vs. T2: 46.93 (10.75); *p* = 0.028; Cohen’s d = −0.29	**Very low** Observational design, serious risks of selection and confounding bias (participation rate ND, potential confounders not considered in analyses), potentially inadequate power
Multi-component COVID-19 prevention program: reorganized wards (e.g., increased ICU beds), procedures (e.g., cleaning and disinfection) and internal paths, increased nurse-to-patient ratios in COVID units, PPE training, other training, promoted participatory approach, autonomy and conscientiousness through continuous clinical and organizational audits, lectures, workshops and meetings, psychological help desk for staff, staff COVID-19 testing (4 months) (Zaghini et al. 2021) [45]	**≠ quality of emotional life** post-program: mean score on emotional subscale of NQoL-SAT-P (SD) that ranges from 1 to 4: T0: 3.13 (.49) vs. T1: 3.16 (.52); *p* = 0.334	**Low** Observational design, risk of confounding bias (several potential confounders ND (i.e., level of adherence to the intervention) or not integrated in analyses (i.e., age, having children))
	**↑ quality of the psychosocial work environment** post-program: mean HSE-MSIT score (SD) on scale of 1 to 5: T0: 2.46 (0.40) vs. T1: 2.32 (0.50); *p* < 0.001	**Low** Observational design, risk of confounding bias (several potential confounders ND (i.e., level of adherence to the intervention) or not integrated in analyses (i.e., age, having children))
	**≠ work demands** (workload, time pressure) post-program: mean HSE-MSIT subscale score (SD) on scale of 1 to 5: T0: 2.81 (0.48) vs. T1: 2.79 (0.58); *p* = 0.601	**Low** Observational design, risk of confounding bias (several potential confounders ND (i.e., level of adherence to the intervention) or not integrated in analyses (i.e., age, having children))
	**↑ job control** post-program: mean HSE-MSIT subscale score (SD) on scale of 1 to 5: T0: 2.76 (0.67) vs. T1: 2.65 (0.65); *p* = 0.020	**Low** Observational design, risk of confounding bias (several potential confounders ND (i.e., level of adherence to the intervention) or not integrated in analyses (i.e., age, having children))
	**↑ managerial support** post-program: mean HSE-MSIT subscale score (SD) on scale of 1 to 5: T0: 2.34 (0.88) vs. T1: 2.17 (0.98); *p* = 0.020	**Low** Observational design, risk of confounding bias (several potential confounders ND (i.e., level of adherence to the intervention) or not integrated in analyses (i.e., age, having children))
	**↑ peer support** post-program: mean HSE-MSIT subscale score (SD) on scale of 1 to 5: T0: 2.12 (0.67) vs. T1: 1.93 (0.69); *p* = 0.001	**Low** Observational design, risk of confounding bias (several potential confounders ND (i.e., level of adherence to the intervention) or not integrated in analyses (i.e., age, having children))
	**↑ quality of relationships at work** (harassment, tension, bullying) post-program: mean HSE-MSIT subscale score (SD) on scale of 1 to 5: T0: 2.23 (0.88) vs. T1: 2.04 (0.68); *p* = 0.001	**Low** Observational design, risk of confounding bias (several potential confounders ND (i.e., level of adherence to the intervention) or not integrated in analyses (i.e., age, having children))
	**≠ role clarity at work** post-program: mean HSE-MSIT subscale score (SD) on scale of 1 to 5: T0: 1.71 (0.52) vs. T1: 1.69 (0.60); *p* = 0.798	**Low** Observational design, risk of confounding bias (several potential confounders ND (i.e., level of adherence to the intervention) or not integrated in analyses (i.e., age, having children))
	**Improvement in how organizational change is managed and communicated at work** post-program: mean HSE-MSIT subscale score (SD) on scale of 1 to 5: T0: 2.98 (0.49) vs. T1: 2.46 (0.79); *p* < 0.001	**Low** Observational design, risk of confounding bias (several potential confounders ND (i.e., level of adherence to the intervention) or not integrated in analyses (i.e., age, having children))
Study reporting on the association between perception of adequate PPE, training and support and mental health indicators 13–25 months after SARS outbreak (no intervention described *per se*) (Maunder et al. 2006) [47]	**20% ↓ likelihood of post-traumatic stress** (IES-R ≥ 26) post-outbreak, multivariate logistic regression model: β = −0.22; *p* = 0.01	**Low** Observational design
	**24% ↓ likelihood of emotional exhaustion** (MBI-EE ≥ 27) post-outbreak, multivariate logistic regression model: β = −0.27; *p* = 0.002	**Low** Observational design
	**Likelihood of psychological distress** (K10 ≥ 16) **not reported** because the “Training, protection and support” indicator was not significant in univariate models	**Low** Observational design

^1^ Detailed descriptions of intervention content and effectiveness are provided in Supplementary Table S1. ^2^ Low: our level of confidence in effect estimates is low, the true effect could be very different from that estimated in the studies; very low: our level of confidence in effect estimates is very low, the true effect is probably very different from that estimated in the studies. ↑ higher; ↓ lower; ≠ no change. AOR: adjusted odds ratio; CI: confidence interval; GAD: Generalized Anxiety Disorder; HADS: Hospital Anxiety and Depression Scale; HSE-MSIT: health and safety executive management standards indicator tool; ICU: intensive care unit; IES-R: impact of event scale-revised; IPC: infection prevention and control; K10: Kessler 10-item psychological distress scale; MBI-EE: Maslach burnout inventory−emotional exhaustion subscale; ND: not documented; NQoL-SAT-P: Nurses Quality of Life Scale−Satisfaction Profile; PHQ-9: Patient Health Questionnaire; PPE: personal protective equipment; PSQI: Pittsburgh sleep quality index; SARS: severe acute respiratory syndrome; SD: standard deviation; UWES-9: Utrecht Work Engagement scale; WEMWBS: Warwick—Edinburgh Mental Wellbeing Scale

## Data Availability

The data presented in this study are available within the article and Supplementary Table S1.

## Supplementary Materials

The following supporting information can be downloaded at: https://www.mdpi.com/article/10.3390/ijerph19159653/s1, Supplementary Table S1. Description of the seven analyzed studies.

## Appendix A

**Table A1:** Medline search strategy combining concepts 1, 2, 3 and 4.

Concept *	Search Strategy
1	(depression or “depressive disorder” or anxiet* or anxious or “mental health” or “mental disorder*” or “adjustment disorder*” or (stress adj3 work*) or distress or ptsd or “post traumatic stress” or “post-traumatic stress” or “vicarious trauma*” or “secondary trauma*” or “compassion fatigue” or “compassion satisfaction” or traumatisation or traumatization or exhaustion or burnout or suicide or suicidal or fear).ti,ab,kw. OR anxiety disorders/ or depressive disorder/ or depressive disorder, major/ or “trauma and stressor related disorders”/ or stress disorders, traumatic/or stress disorders, post-traumatic/ OR emotions/ or bereavement/ or sadness/ or grief/ or guilt/ or loneliness/ or psychological distress/ or sadness/ OR exp adaptation, psychological/ or exp stress, psychological
2	((interven* or program* or initiative* or approach* or project* or strateg* or reorganis* or reorganiz* or “re-organis*” or “re-organiz*” or redesign or “re-design” or restructuring or re-structuring or policy or policies or regulation* or guidance or guideline or standard or solution or change) adj5 (workplace or worker* or “work-place” or “workplace based” or “work-place based” or workload or workflow* or staff or personnel or employee* or occupation* or industry or “public sector” or “private sector” or employer or organization* or organisation* or task* or colleague* or coworker* or co-worker* or supervisor* or manager* or corporate or corporation or “iso-strain” or ((quantitative or mental or emotional or psychological) adj1 (demand* or workload)) or (job adj1 (control or demands or strain)) or “psychological strain” or “stress at work” or “stressful working condition” or “emotionally demanding work” or (decision* adj1 (latitude or authority or autonomy)) or (skill adj (discretion or utili#ation)) or “effort-reward” or “((social or corporate or organizational or organisational or company) adj1 (justice or leadership or trust))” or “team spirit” or harassment or violence or bullying or ((colleague* or coworker* or co-worker* or supervisor* or superior* or manager* or management) adj1 support) or ((corporate or safety or psychosocial) adj1 (climate or culture or environment)) or “flexible working conditions” or “work-life balance” or “work life balance” or “work-life conflict” or “work life conflict” or (work adj2 family) or “moral dilemma” or “moral injury” or “ethical dilemma” or “management practice*” or “corporate management” or “workplace management” or “work place management” or communication or transparency or purposeful)).ti,ab,kw. OR ergonomics/ or man-machine systems/ or organizational innovation/ OR Organizational culture/
3	(efficien* or inefficien* or effective or efficacy or ineffective or evaluat* or assess*).ti,ab,kw. or ((intervention adj2 (trial* or study or studies)) or “Before and After Stud*” or “Before-After Stud*” or (pre adj5 post) or survey or surveys or questionnaire* or “focus group*” or interview*).ti,ab,kw. or comparative effectiveness research/ OR evaluation studies as topic/ OR program evaluation/ OR intervention studies/ OR Controlled Before-After Studies/ or (“Evaluation studies”).pt.
4	(H1N1 OR “middle east respiratory syndrome*” OR MERS OR SARS* OR “severe acute respiratory syndrome*” OR “SARS-CoV-2” OR “SARS-CoV” OR “COVID” OR “COVID-19” OR coronavirus* or pandemic* or epidemic* or influenza or flu or outbreak* or ebola or ebolavirus or zika or quarantine or confinement or ((health or sanitar*) adj1 (crisis or crises or emergenc*))).ti,ab,kw. Or COVID-19/ or epidemics/ or pandemics/ or disease outbreaks/

* Concept 1: “mental health problems”; concept 2: “organizational or psychosocial work environment interventions”; concept 3: “effectiveness evaluation”; concept 4: “epidemic/pandemic”. Natural language terms are the same for all databases. Database-specific terms, ending with an oblique symbol (/), can be provided for the other databases upon request to the authors.

**Table A2:** Methodologic quality assessment instrument: items and scoring.

Item	Scoring (Number of Points)
**Was the research question or study objectives clear and explicitly stated?**	
No research question or study objective was described	0
A research question or study objective was mentioned but was not clear	1
The research question and/or study objectives were clear and explicitly stated	2
**Did the study include a control group?**	
There was no control group	0
There was a control group, but it was not appropriate	1
There was an appropriate control group	2
**Were study participants randomly assigned to the control or intervention group? If study participants were not randomly assigned, were workers’ baseline sociodemographic, occupational exposure and mental health outcome characteristics measured?**	
Study participants were not randomly assigned to the control or intervention group and their baseline characteristics were not measured	0
Study participants were not randomly assigned to the control or intervention group but some of their baseline characteristics were measured (however, important baseline sociodemographic, occupational exposure or health characteristics are missing)	1
Study participants were randomly assigned to the control or intervention group OR baseline sociodemographic, occupational exposure and health characteristics were measured	2
**Were relevant occupational exposures measured before (at baseline) and after (at follow-up) the intervention?**	
Relevant occupational exposures were not measured	0
Some very relevant occupational exposures were not measured	1
Relevant occupational exposures were measured either only at baseline or at follow-up, but not at both time points	1
Relevant occupational exposures were measured at baseline and at follow-up, but not in the same participants (unpaired data)	1
Relevant occupational exposures were measured at baseline and at follow-up in the same participants (paired data)	2
**Were occupational exposure measures appropriate, valid, reliable and sensitive to change?**	
Occupational exposure measures were not appropriate	0
Occupational exposure measures seem appropriate, but there was no confirmation that they were valid, reliable and/or sensitive to change	1
Occupational exposure measures were appropriate, valid, reliable and sensitive to change	2
**Was the mental health outcome measured before (at baseline) and after (at follow-up) the intervention?**	
A mental health outcome was not measured	0
The mental health outcome was measured either only at baseline or at follow-up, but not at both time points	1
The mental health outcome was measured at baseline and at follow-up, but not in the same participants (unpaired data)	1
The mental health outcome was measured at baseline and at follow-up in the same participants (paired data)	2
Not applicable: study objective is to measure the effect of the intervention on occupational exposures, not mental health	1
**Was the mental health outcome measure appropriate, valid, reliable and sensitive to change?**	
The mental health outcome measure was not appropriate	0
The mental health outcome measure seems appropriate, but there was no confirmation that it was valid, reliable and/or sensitive to change	1
The mental health outcome measure was appropriate, valid, reliable and sensitive to change	2
Not applicable: study objective is to measure the effect of the intervention on occupational exposures, not mental health	1
**Was the length of follow-up after the end of implementation of the intervention appropriate?**	
The length of follow-up after the end of implementation of the intervention was not indicated	0
The follow-up was done before the end of intervention implementation or the length of follow-up was too short to allow for an effect on the health outcome (or on another measured outcome) to be demonstrated	1
The length of follow-up after the end of implementation of the intervention was appropriate	2
**Was study participation rate after recruitment documented and adequate for the experimental and control groups?**	
Study participation rate after recruitment was not documented or was <60%	0
Study participation rate after recruitment was between 60 and 79%	1
Study participation rate after recruitment was ≥80%	2
**Was the loss of study participants to follow-up in the experimental and control groups acceptable?**	
The loss to follow-up was not documented or was >30%	0
The loss to follow-up was between 21 and 30%	1
The loss to follow-up was ≤20%	2
**Were the participants who dropped out of the study (drop-outs) comparable to those who completed the study (completers)?**	
A comparison of the characteristics of drop-outs and completers was not documented	0
There were important differences in the characteristics of drop-outs and completers, but this was not considered in the analyses	1
There were no important differences in the characteristics of drop-outs and completers, and this was documented OR the loss to follow-up was ≤20%	2
**Was the implementation of intended changes documented and were changes implemented as intended?**	
The implementation of changes was not documented	0
The implementation of changes was documented but they were not implemented or only some intended changes were implemented	1
The implementation of changes was documented and the majority of intended changes were implemented	2
**Were potential confounders of the effect of the intervention on the mental health outcome (ex. history of mental illness, intervention compliance) and on the work exposures measured considered and properly taken into account in the analysis (ex. adjustment, stratification) or interpretation of results?**	
No potential confounders were measured	0
Important confounders were not measured or measured confounders were not properly taken into account in the analysis or were only considered in interpretation of results	1
Potential confounders were measured and properly taken into account in the analysis	2
**Were contextual factors and co-interventions that could influence the results considered in the analysis or in the interpretation of the results?**	
No contextual factors or co-interventions that could influence the results were documented	0
Only a few relevant contextual factors or co-interventions were documented or considered in the analysis or in the interpretation of the results	1
Relevant contextual factors and co-interventions were documented and considered, either in the analysis or in the interpretation of the results	2
**Was the statistical analysis appropriate for measuring the effectiveness of the intervention?**	
The analysis was inadequately described, precluding us from evaluating its appropriateness or the analysis was inappropriate	0
The statistical power of the study or at least one other important element of analysis was inappropriate	1
The analysis and power of the study were appropriate	2
