# Supplementary material for: Are Organizational Interventions Effective in Protecting Healthcare Worker Mental Health during Epidemics/Pandemics? A Systematic Literature Review
Source: Int J Environ Res Public Health. 2022 Aug 5;19(15):9653. doi: 10.3390/ijerph19159653 (PMC9368524; doi:10.3390/ijerph19159653)
Supplement: Supplementary file 1 [file ijerph-19-09653-s001.zip › ijerph-1835811-supplementary.pdf]

Supplementary Table S1. Description of the seven analyzed studies

Study (country)	Design	Participants analyzed	Participation rate at recruitment (%)	Loss to follow-up rate (%)	Intervention content & implementation	Intervention duration & timing of follow-up	Work exposures measured (physical, biological &/or psychosocial)	Mental health & related outcomes measured	Effect of intervention on work exposures	Effect of intervention on mental health & related outcomes
Beneria et al., 2020 (Spain)	Prospective cohort with control group	I: 77 healthcare workers in a tertiary hospital receiving COVID-19 patients (49% doctors, 51% nurses), who followed simulation-based teamwork training before the pandemic (70% F)<br><br>C: 64 healthcare workers (45% doctors, 55% nurses) from the same hospital who had never received simulation training, chosen by their colleagues in the I group (84% F)	I: 80% (77/96)<br><br>C: 48% (64/134)	ND<br>(but there seems to be no missing data in study Table 1)	Intervention content:<br>• 25-hour course on simulation-based teamwork training to develop leadership, communication, briefing/debriefing skills & to become a simulation instructor; 13 hours on-line, 12 hours practical training on-site, given between Nov. 2019 & March 2020 by the Vall Hebron Advanced Clinical Simulation centre<br><br>Implementation/usage:<br>• 3 groups of 32 workers followed the training, of whom 9% had already completed a simulation instructor course prior to the intervention	Duration:<br>• 25 hours between Nov. 2019 & March 2020<br><br>Follow-up:<br>• 2-6 months later, in May 2020	Biological exposures<br>• Self-reported contact with COVID-19-positive patients<br>o Daily<br>o >1 day/week<br>o <5 days/month<br>o Rarely<br><br>• Self-reported work in a COVID-19 area (yes/no)	Anxiety & depression HADS>12	ND	• Level of anxiety & depression, mean HADS (SD):<br>o I: 14.23 (7.41)<br>o C: 12.08 (6.66)<br>o Mean difference = −2.15 (95% CI: -4.52 – 0.22)<br><br>• Prevalence of anxiety & depression, % HADS>12: I: 54.6 vs. C: 42.2; p = 0.346<br><br>• Logistic regression analysis of the association between participating in the training & probability of HADS>12:<br>o Odds ratio for participating in the training not provided in Table 2<br>o Unclear if all covariables are included in analysis (age, sex, having minors in charge, married, profession, contact with COVID patients)<br><br>• Stratified regression analyses according to working in a COVID-19 area/having had contact with COVID-19 patients (unclear which exposure indicator is used, contradiction between text p.3 and title of Table 3):<br>o For workers having had contact with COVID-19 patients (n=88), participation in the training (vs. no training) increased the probability of

Study (country)	Design	Participants analyzed	Participation rate at recruitment (%)	Loss to follow-up rate (%)	Intervention content & implementation	Intervention duration & timing of follow-up	Work exposures measured (physical, biological &/or psychosocial)	Mental health & related outcomes measured	Effect of intervention on work exposures	Effect of intervention on mental health & related outcomes
										HADS>12: AOR (95% CI): 2.56 (1.03 – 6.36); p=0.043<br>o For workers not having had contact with COVID-19 patients, AOR not provided in Table 3.<br><br>Authors were contacted to clarify their results on June 14, 2021. No reply.
Blake et al., 2020 (United Kingdom)	Cross-sectional, no control group, but those who accessed the centres were compared to those who did not	819 employees of an acute hospital trust: 46% nursing staff, 21% paramedical, scientific & technical staff, 18% administrative staff; 37% with line manager responsibilities (88% F)	<5% (819/18,011)<br><br>Authors state that sample is representative of hospital trust employees (p.7), but no data is provided	N/A	Intervention content:<br>• Two wellbeing centres (1 purpose-built wellbeing room & 1 converted hospital ward) equipped with wellbeing buddies trained in psychological first aid (listening, comforting and guiding to information, services & support); wellbeing buddies are 134 employees with reduced workload because of pandemic, who volunteered for the role and received training and supervision by two clinical psychologists (10 group sessions of 2-hour training; 11 supervision sessions of 1 hour); no qualifications required prior to training. Wellbeing buddies ensured room cleanliness & respect of health & safety measures. They worked in pairs and committed to a minimum 4-hour	Duration:<br>• ~4 months, centres opened April 6, 2020<br><br>Follow-up:<br>• Centre usage data collected by wellbeing buddies over 17 weeks, April-July (peak of 1st wave)<br><br>• Health data collected by on-line questionnaire for 6 weeks between July & August 2020	Biological exposure<br>• Self-reported work in COVID-19 high-risk area (COVID-19 wards, ICU, emergency department, residential home, meet & greet, other self-defined high-risk area) vs. low-risk area (mainly working from home)	• Mental wellbeing (14-item WEMWBS; score 14-70, higher scores indicate higher wellbeing, WEMWBS≤40 considered poor wellbeing)<br><br>• Work engagement, considered the antipode of burnout [3 items of dedication subscale of UWES-9; “I am enthusiastic about my job”, “My job inspires me”, “I am proud of the work I do”, 0-never to 6-always/every day, total score 0-6, engagement	ND	• Wellbeing is higher among centre users than non-users, mean (SD): 47.04 (9.49) vs. 45.11 (9.35); p = 0.02<br><br>• Work engagement is high & tends to be higher among centre users than non-users, mean (SD): 5.02 (1.38) vs. 4.83 (1.15); p = 0.08<br><br>• Proportion (%) of centre users and non-users reporting presenteeism in previous 12 months is similar:<br>no, never: 16.31 vs. 14.97<br>yes, once: 17.05 vs. 12.76<br>yes, 2 to 5 times: 16.92 vs. 12.64<br>yes, >5 times: 4.53 vs. 4.41<br>p = 0.28<br><br>• Proportion (%) of centre users and non-users reporting intention to quit is similar: 16.31 vs. 15.09; p = 0.25<br><br>No multivariate analyses: comparisons do not adjust for sociodemographic characteristics & work in a COVID-19 high- vs. low-risk

Study (country)	Design	Participants analyzed	Participation rate at recruitment (%)	Loss to follow-up rate (%)	Intervention content & implementation	Intervention duration & timing of follow-up	Work exposures measured (physical, biological &/or psychosocial)	Mental health & related outcomes measured	Effect of intervention on work exposures	Effect of intervention on mental health & related outcomes
					shift in the centres, with some doing 2 shifts/day, multiple times/week. Centres open 8am-8pm, 7 days/week, but reduced hours on week 9 (Monday-Friday, 10am-4pm), based on usage data and buddies resuming normal work activities. Refreshments and, for a limited time early on, charitable donations for employees were available in the centres (ex. care packages, toiletries, snacks, washable uniform bag).<br>Implementation/usage:<br>• 219-2,605 visits/week over 17 weeks April-July 2020, peak during week of April 20th with subsequent decrease<br>• 94% of respondents were aware of centres<br>• 55% of respondents used them<br>• 53 "wobbles", i.e. active seeking of emotional support during a visit (0.3% of visits)<br>• High level of satisfaction with the centres, mean score (SD) 8.15 (2.27) on scale of 1-10<br>• Human resource & financial investment of ~16,000£ deemed unsustainable by the authors (includes human resources &			level very low (<1.33), low (1.34–2.90), moderate (2.91–4.70), high (4.71–5.69), very high (≥5.70)]<br>• Presenteeism past 12 months (1 item: "As far as you can recall, has it happened over the previous 12 months that you have gone to work despite feeling that you really should have taken sick leave due to your state of health?"<br>1 = no, never<br>2 = yes, once<br>3 = yes, 2 to 5 times<br>4 = yes, more than 5 times)<br>• Intention to quit (1 item: "Are you considering leaving your job?" yes/no)		area. But centre users differed from non-users : centre users were more likely to be younger (21-30 years old), in nursing professions and working in medical & surgical departments compared to non-users, least likely to work in maintenance or general management or to be doctors in training/clinical fellows and ambulance workers, see study Table 5; work engagement was higher among those who accessed the centre and those working in COVID-19 high-risk areas, see study Table 3

Study (country)	Design	Participants analyzed	Participation rate at recruitment (%)	Loss to follow-up rate (%)	Intervention content & implementation	Intervention duration & timing of follow-up	Work exposures measured (physical, biological &/or psychosocial)	Mental health & related outcomes measured	Effect of intervention on work exposures	Effect of intervention on mental health & related outcomes
					costs for set-up, furniture, cleaning supplies and training/supervision services by clinical psychologists)<br>• Self-reported barriers to use included breaks not long enough or unable to take a break, preferring to take private break, no buddy available, insufficient space or seating, centre too far from work area, belief that frontline staff should be prioritized to use the centres, unaware if able to attend or if non-clinical staff can attend, not feeling sanctioned by manager to use the centres, technical issue with magnetic card blocking access to one of the sites weeks 1-3<br>• Provision of beverages in the centres was a key reported benefit. The literature suggests that healthcare staff dehydration is prevalent, related to working in warm environments and missing breaks, which impacts cognitive function and performance. Authors relayed anecdotal reports of staff avoiding to drink in order not to waste PPE during the pandemic.					

Study (country)	Design	Participants analyzed	Participation rate at recruitment (%)	Loss to follow-up rate (%)	Intervention content & implementation	Intervention duration & timing of follow-up	Work exposures measured (physical, biological &/or psychosocial)	Mental health & related outcomes measured	Effect of intervention on work exposures	Effect of intervention on mental health & related outcomes
Chen et al., 2006 (Taiwan)	Before-after, no control group	116 nurses from the largest obligatory SARS designated treatment hospital in Taiwan (Ho-Ping Hospital) (98% F)	Unknown (120/? eligible)	3%	Intervention content:<br>• In May 2003, the government of Taiwan designated a community hospital as an obligatory SARS center in order to limit community transmission. The hospital initiated a systematic SARS prevention programme (inspired by CDC and WHO recommendations) comprising:<br>o Weekly unit-based assignments, 3-4 SARS units, while other units rest<br>o Daily hours worked limited to 8 hours to prevent fatigue<br>o Staffing adjustments according to number of admitted SARS patients to prevent staff shortages or surplus<br>o Daily information updates	Duration:<br>• 3 months<br><br>Follow-up:<br>• T0: before start of SARS patient care<br>• T1: 2 weeks after caring for SARS patients under the prevention programme<br>• T2: 1 month after start of programme & patient care<br>• T3: 1 month following return to normal (hospital no longer designated SARS centre), i.e. 3 months of	Psychosocial exposure<br>• Belief that current protective equipment is sufficient (yes/no)	• Anxiety: Chinese translation of 20-item Zung self-rating anxiety scale (SAS)<br><br>• Depression: Chinese translation of 20-item Zung self-rating depression scale (SDS) For both scales, levels are categorized as 50–59 mild 60–69 moderate ≥70 severe<br><br>• Sleep quality (PSQI) ≤5 good >5 poor	ND	Anxiety levels, mean SAS score (SD)<br>• T0: 60 (9.28) Moderate<br>• T1: 51 (10.32) Mild<br>• T2: 50 (9.84) Mild<br>• T3: 46 (7.48) No anxiety<br><br>Depression levels, mean SDS score (SD)<br>• T0: 61 (12.62) Moderate<br>• T1: 51 (11.94) Mild<br>• T2: 50 (10.60) Mild<br>• T3: 48 (10.76) No depression<br>Sleep quality, mean PSQI (SD)<br>• T0: 12 (3.83) Poor<br>• T1: 10 (3.43) Poor<br>• T2: 10 (3.77) Poor<br>• T3: 8 (2.75) Poor<br><br>Changes in anxiety, depression & sleep quality levels 2 weeks, 1 month & 3 months under the programme, generalized estimating equation models adjusted for temporal variation, perceived stress level before the pandemic and level of family support while caring for patients:

Study (country)	Design	Participants analyzed	Participation rate at recruitment (%)	Loss to follow-up rate (%)	Intervention content & implementation	Intervention duration & timing of follow-up	Work exposures measured (physical, biological &/or psychosocial)	Mental health & related outcomes measured	Effect of intervention on work exposures	Effect of intervention on mental health & related outcomes
					o Supplements made available to staff to strengthen their immune system<br>o IPC measures & protocols for the handling of SARS patients: negative pressure isolation rooms, protocols for room entry & exit, coordination of treatments to minimize room traffic, protocols for personal hygiene & for room/equipment disinfection (e.g. UV light), protocols for PPE donning & doffing, with indication to wear 2 layers (N95 & surgical mask) & conduct regular N95 seal checks<br>o Availability of the latest PPE: scrub suits, isolating dresses, surgical caps, sterilized gloves, foot wraps, N95 masks, surgical masks, P100 masks, and safety glasses<br>o In-service training: 53 classes on SARS patient care and IPC measures, including use of PPE<br>o A multidisciplinary mental health team (psychiatrists, social workers, psychological counselors, psychiatric nurses) available to patients and medical staff. Nursing staff	SARS patient care				Changes in anxiety levels (improvement)<br>• T0 vs. T1: Z = -2.68; p = 0.0075<br>• T0 vs. T2: Z = -4.45; p<0.0001<br>• T0 vs. T3: Z = -6.58; p<0.0001<br>Changes in depression levels (improvement)<br>• T0 vs. T1: Z = -4.58; p<0.0001<br>• T0 vs. T2: Z = -4.80; p<0.0001<br>• T0 vs. T3: Z = -6.37; p<0.0001<br>Changes in sleep quality (improvement)<br>• T0 vs. T1: Z = -2.79; p = 0.0053<br>• T0 vs. T2: Z = -3.14; p = 0.0017<br>• T0 vs. T3: Z = -3.37; p = 0.0008<br><br>Some of the other covariables presented by the authors seem to not have been integrated in models because they were not associated with anxiety, depression or sleep quality, but this is not completely clear (age, sex, education level, marital status, current professional title, religious affiliation, having volunteered to care for SARS patients). A few potential confounders were not measured (level of compliance with the programme, medication use for anxiety or depression - sleep medication use was assessed in the PSQI, other factors outside of work, changing work

Study (country)	Design	Participants analyzed	Participation rate at recruitment (%)	Loss to follow-up rate (%)	Intervention content & implementation	Intervention duration & timing of follow-up	Work exposures measured (physical, biological &/or psychosocial)	Mental health & related outcomes measured	Effect of intervention on work exposures	Effect of intervention on mental health & related outcomes
					identified patients to be called by the mental health team daily. A mental health clinic was opened for healthcare workers<br>Implementation/usage:<br>• Programme & measures are said to have been deployed, but no details on how these were applied, nor on levels of worker compliance<br>• Authors mention in discussion that prolonged wearing of PPE (6-8 hours) precluded eating, drinking or using the bathroom and that some staff became dehydrated & developed skin rashes. But whether & how this affected compliance is not reported.					schedules mentioned in discussion).
Giordano et al., 2021 (Italy)	Before-after pilot study, no control group	17 healthcare leaders & 62 of their staff (coordinators, nurses, care assistants, educators, etc.) drawn from a social & community enterprise providing healthcare services, education & social assistance to elderly people & at-risk minors & mothers in the	Unknown (21 leaders & 62 of their staff/? eligible)<br><br>Leader participation was voluntary following an invitation to a virtual presentation of the study and curriculum. Leaders were invited to inform their staff about their participation in the programme	19% of leaders & 0% of their staff were lost to follow-up	Intervention content:<br>• Phase 1 pilot implementation of the "R2 for Leaders" resilience programme tailored for healthcare leaders dealing with COVID-19. The 2 R's stand for individual "rugged" qualities & external "resources". The curriculum is intended to equip healthcare leaders to better lead their staff & organizations during the pandemic. Specifically, it aims to help participants: 1)	Duration:<br>• 12 weeks<br><br>Follow-up:<br>• T1: April 2020 (1 week before programme start)<br>T2: July 2020 (1 week after programme end)	Biological exposure<br>• Self-reported number of suspected or confirmed COVID-19 patients encountered, measured at T1<br><br>Psychosocial exposure<br>• Modified Italian version of the HSE-MSIT measuring the psychosocial work environment (authors refer to it as "Work-related	• Professional burnout, 2 items from emotional exhaustion subscale, MBI-EE: "I feel burned out from my work" (1-Never to 5-Every day) & "I feel like I'm burning out from my work" (1-Not at all to 5-A lot). Authors state that	• No difference in leaders' mean HSE-MSIT scores (SD) T1: 50.50 (15.33) vs. T2: 50.56 (15.17) $t(16) = -0.44$, $p = 0.966$<br><br>• Relatively small statistically significant decrease in staff mean HSE-MSIT	• Relatively small statistically significant decrease in leaders' mean (SD) MBI-EE scores between T1: 6.31 (1.35) & T2: 5.37 (1.20) $t(16) = 2.61$, $p = 0.020$ Hedge's g (corrected Cohen's *d* for small samples <50) = −0.30<br><br>• No clinically meaningful nor statistically significant decrease difference in staff mean (SD) MBI-EE scores between T1: 4.70 (1.63) & T2: 4.35 (1.64) $t(60) = 1.68$, $p = 0.098$

Study (country)	Design	Participants analyzed	Participation rate at recruitment (%)	Loss to follow-up rate (%)	Intervention content & implementation	Intervention duration & timing of follow-up	Work exposures measured (physical, biological &/or psychosocial)	Mental health & related outcomes measured	Effect of intervention on work exposures	Effect of intervention on mental health & related outcomes
		province of Bergamo, Italy (85% F)	and invite them to take part in the assessment survey as beneficiaries of the programme on a voluntary basis.		Identify practices that support organizational & healthcare worker resilience; 2) Learn how to help staff navigate to, and negotiate for, resources in order to support mental health and wellbeing inside & outside the workplace; 3) Implement a methodology to build a resilience-based prevention program that is context-specific. Curriculum consisted of 12 virtual 2-hour weekly sessions over 3 months given in parallel to a group of elderly care leaders and child protection & childcare coordinators. 2 initial participatory needs analysis sessions were held with leaders on challenges (ex. loneliness, ineffectiveness) & sources of resilience (ex. peer & intimate relationships, skills, sense of personal agency, organizational culture), akin to a Delphi process, consisting of personal reflections and small & whole-group reporting. 10 subsequent sessions were held on promising practices for prevention focused on each of the 10 identified elements of resilience (see study		Stress"). 5-point Likert-type response scale, lower scores reflect worse exposure. Authors excluded 13 items from the original 35-item scale, for a total of 22 items and score range 22-110 (authors were contacted & confirmed number of items and score range on Nov. 17, 2021).	scores range from 5 to 10, but reported results are outside this range.	scores (SD) (authors interpret as improvement, i.e. reduced work-related stress on p.11) T1: 50.18 (10.56) vs.<br>T2: 46.93 (10.75)<br>$t(60) = 2.25$, $p = 0.028$<br>Cohen's $d = -0.29$<br><br>Paired-samples t-tests. Authors used Cohen's standards to interpret intervention effect magnitude as large (0.8), medium (0.5) & small (0.2).	Paired-samples t-tests. Authors used Cohen's standards to interpret intervention effect magnitude as large (0.8), medium (0.5) & small (0.2).<br><br>Analyses also showed meaningful improvements in resilience (individual & external resources to deal with adversity) & self-efficacy in both leaders and their staff.<br><br>Potential confounders not considered in analyses. Authors discuss that negative results could partly be explained by the limited time staff were in contact with supervisors during the pandemic, or lack of opportunities during staff meetings to reflect on recovery. Participants also reported feeling less engaged during last sessions due to exhaustion after first pandemic months, which could explain reduced programme effectiveness on exhaustion scores.

Study (country)	Design	Participants analyzed	Participation rate at recruitment (%)	Loss to follow-up rate (%)	Intervention content & implementation	Intervention duration & timing of follow-up	Work exposures measured (physical, biological &/or psychosocial)	Mental health & related outcomes measured	Effect of intervention on work exposures	Effect of intervention on mental health & related outcomes
					Table 1). Sessions had five parts: (i) for each element, there was an initial supervision on the support healthcare leaders provided to their teams during remote staff meetings, based on the previous R2 sessions; (ii) this was followed by a group reflection triggered by an inspirational video, photo or narration to identify whether and how the target resources were a source of resilience for leaders and their staff; (iii) session workshops to facilitate self-expression, shared meaning making and mastery of both suffering and resilience; (iv) a group discussion in which healthcare leaders proposed different ways to introduce the target resource in the staff meetings (with consideration to time limitations); (v) lessons learnt from the session. During the sessions, examples of tasks leaders could implement were given, i.e. creating a self-care plan for the whole team, thanking workers, avoiding top-down solutions, clearly defining duties &					

Study (country)	Design	Participants analyzed	Participation rate at recruitment (%)	Loss to follow-up rate (%)	Intervention content & implementation	Intervention duration & timing of follow-up	Work exposures measured (physical, biological &/or psychosocial)	Mental health & related outcomes measured	Effect of intervention on work exposures	Effect of intervention on mental health & related outcomes
					responsibility in their teams, encouraging staff to ask for help.<br>Implementation/usage:<br>• Leaders attended at least 85% of group sessions.<br>• High rates of general perceived programme usefulness (mean score of 2.5 on 3 points; SD = 0.52).<br>• Perceived domains of acquired knowledge included skills related to interaction & dialogue with their staff (mean of 2,42 on 3; SD = 0.51), strengthened personal resources (mean of 2,58 on 3; SD = 0.67) & team resources (mean of 2,5 on 3; SD = 0.52).<br>• Open-ended comments reported specific benefits, e.g. improvement in interpersonal relationships with other leaders & growth in self-awareness.<br>• Participants suggestion for programme improvement: reduce number of sessions given the level of commitment required during a very stressful period. Participants reported feeling less engaged during last sessions due to physical & emotional fatigue after first months of the pandemic.					

Study (country)	Design	Participants analyzed	Participation rate at recruitment (%)	Loss to follow-up rate (%)	Intervention content & implementation	Intervention duration & timing of follow-up	Work exposures measured (physical, biological &/or psychosocial)	Mental health & related outcomes measured	Effect of intervention on work exposures	Effect of intervention on mental health & related outcomes
Maunder et al., 2006 (Canada)	Cross-sectional, with control group (used to compare psychological impact of SARS in workers who treated SARS patients or not in hospitals with same SARS precautions)	587 nurses from medical & surgical inpatient units & all staff from ICU, emergency departments & SARS isolation units of 9 Toronto hospitals having treated SARS patients, 40 clinical units in total (86% F)<br><br>C: 182 healthcare workers from 4 Hamilton hospitals that experienced the same processes & precautions associated with province's SARS response (e.g. protocols for staff screening, isolation procedures) but did not have SARS patients, 15 clinical units in total (90% F)<br><br>74% nurses, 3% doctors (for the total sample n=769)	39% (769/1,984)<br><br>After the main study, a representativeness survey was conducted between September & November 2005 with 255 Toronto healthcare workers (response rate 99%); study participants were found to be similar to non-participants with respect to age, job title, years of healthcare experience & perceived impact of the epidemic on their life, but those who had contact with SARS patients were overrepresented among participants compared to non-participants	N/A	Intervention content:<br>• An intervention *per se* is not described. Authors are evaluating the association between mental health outcomes and the following 9-item indicator, measuring perceived adequacy of PPE, training, emotional & practical support in the workplace ("Training, protection & support" indicator):<br>1) I had adequate training to deal with the situations that I faced;<br>2) Infection control procedures were adequately explained;<br>3) I received adequate training in infection control procedures;<br>4) I was provided with the protective equipment and procedures that I needed;<br>5) I had someone to ask when I had problems using equipment;<br>6) The hospital where I worked took my well-being into account when decisions were	Duration:<br>• ~8-9 months, covering the period of the SARS outbreak, February-fall 2003 (day last SARS patient was discharged or deceased)<br><br>Follow-up:<br>• ~1-2 years (13-25 months, median 19 months) after end of SARS epidemic in Toronto hospitals, between October 23, 2004 & September 30, 2005	Psychosocial exposure<br>• Job Stress, 5 items:<br>1) There was more conflict among colleagues at work<br>2) I felt more stressed at work<br>3) I had to do work that I normally don't do<br>4) I had an increase in workload<br>5) I had to work overtime<br><br>Biological exposures<br>• Worked on SARS unit (<5 shifts vs. $\geq$5 shifts)<br>• Worked in ICU (<5 shifts vs. $\geq$5 shifts)<br>• Worked in emergency (<5 shifts vs. $\geq$5 shifts)<br>• Ever in SARS patient room (yes/no)<br>• Touched SARS patient (yes/no)<br>• Protected contact with saliva or	• Post-traumatic stress, IES$\geq$26<br><br>• Psychological distress, K10$\geq$16<br><br>• Professional burnout, emotional exhaustion subscale, MBI-EE$\geq$27<br><br>• Increase in smoking, alcohol consumption, use of non-prescription medication or activities that could interfere with work or relationships since the SARS epidemic<br><br>• $\geq$4 work shifts missed since the SARS epidemic in the 4 months preceding the survey because of stress, illness or fatigue	ND	• Perceived adequacy of PPE, training & workplace support was associated with reduced likelihood of post-traumatic stress (β = -0.22; p = 0.01) & emotional exhaustion (β = -0.27; p = 0.002) in multivariate logistic regression models adjusted for maladaptive coping style regarding SARS. Other covariables were not included in final models because NS (p$\geq$0.05) in univariate analyses (e.g. Job stress, attachment anxiety, occupation, work in ICU, unprotected contact with SARS patient, etc.).<br><br>• The association is not presented for psychological distress by study authors because the "Training, protection & support" indicator was NS in univariate models.<br><br>• In a subsequent publication on a subsample of the same healthcare workers (n=139) from Toronto hospitals (Lancee et al., 2008), authors report a protective effect of perceived adequacy of PPE, training & workplace support on incidence of a psychiatric diagnosis 1-2 years after the

Study (country)	Design	Participants analyzed	Participation rate at recruitment (%)	Loss to follow-up rate (%)	Intervention content & implementation	Intervention duration & timing of follow-up	Work exposures measured (physical, biological &/or psychosocial)	Mental health & related outcomes measured	Effect of intervention on work exposures	Effect of intervention on mental health & related outcomes
					made that affected me;<br>7) Emotional support (e.g. counseling) was available to those who needed help;<br>8) I felt appreciated by the hospital/clinic/my employer;<br>9) My hospital/workplace was supportive<br>Implementation/usage:<br>• ND		phlegm of SARS patient (yes/no)<br>• Unprotected exposure to SARS patient (yes/no)<br>• In SARS patients' rooms >5min. or >5 times (yes/no)<br>• Quarantined (never, ≤10 days, >10 days)	• Decrease in hours worked since the SARS epidemic<br>• Decrease in face-to-face patient contact since the SARS epidemic		epidemic (β = -0.20; p = 0.03), in a logistic regression analysis adjusting for history of psychiatric illness & years of healthcare work experience.
Zaghini et al., 2021 (Italy)	Before-after, no control group	322 nurses directly caring for SARS-CoV-2-positive patients in a university hospital transformed into a COVID-designated hospital (76% F)	At least 322/350 = 92%<br><br>Exact number responding at T0 not specified	At most 8%<br><br>322 complete questionnaires at T0 & T1 on 350	Intervention content:<br>Before the arrival of COVID patients in the hospital:<br>• Reorganized wards (e.g. increased ICU beds), procedures (e.g. cleaning and disinfection) & internal paths (to separate SARS-CoV-2-positive from -negative patients)<br>• Increased nurse-to-patient ratios from 1:9 to 1:6 in COVID units of medium-intensity care and from 1:4 to 1:2 in high-intensity care units, maintaining these ratios over 24 hours<br>• Aimed to increase nurses' clinical knowledge and competence regarding SARS-CoV-2, including training on the correct use of individual protection devices & creation of	Duration:<br>• 4 months, starting on March 2, 2020<br><br>Follow-up:<br>• T0: Feb. 19-29, 2020 (before intervention)<br>T1: July 6-19, 2020	Psychosocial exposures<br>• HSE-MSIT, 35 items measuring the psychosocial work environment & its components (7 subscales) (authors call it "Work-related Stress"), 5-point Likert-type response scale (1 "Never" to 5 "Always"), higher scores reflect worse exposure:<br>1) Demands (workload, time pressure)<br>2) Control<br>3) Managerial Support<br>4) Peer Support<br>5) Relationships (harassment, tension, bullying)<br>6) Role clarity	Quality of emotional life measured with emotional subscale of Nurses Quality of Life Scale - Adjustment of Satisfaction Profile (NQoL-SAT-P); assesses level of satisfaction with respect to 8 items (1 "Very dissatisfied" to 4 "Very satisfied"): resistance to stress, affective tone, mental efficacy, emotional stability, self-confidence, problem solving skills, psychological autonomy, self-control	Mean HSE-MSIT score (SD)<br>• Overall psychosocial risk ("work-related stress")<br>T0: 2.46 (.40)<br>T1: 2.32 (.50)<br>t = 4.42<br>p<0.001<br><br>• Demands<br>T0: 2.81 (.48)<br>T1: 2.79 (.58)<br>t = 0.52<br>p = 0.601<br><br>• Control<br>T0: 2.76 (.67)<br>T1: 2.65 (.65)<br>t = 2.35<br>p = 0.020<br><br>• Managerial Support<br>T0: 2.34 (.88)<br>T1: 2.17 (.98)<br>t = 2.34<br>p = 0.020	Mean score on emotional subscale of NQoL-SAT-P (SD)<br>• T0: 3.13 (.49)<br>• T1: 3.16 (.52)<br>t = -0.97<br>p = 0.334<br><br>Paired sample t-test.<br>Result does not consider the level of adherence to the intervention, nor other potential confounders like age & having children.

Study (country)	Design	Participants analyzed	Participation rate at recruitment (%)	Loss to follow-up rate (%)	Intervention content & implementation	Intervention duration & timing of follow-up	Work exposures measured (physical, biological &/or psychosocial)	Mental health & related outcomes measured	Effect of intervention on work exposures	Effect of intervention on mental health & related outcomes
					online content available to all staff (e.g. reporting guidelines, updated government information)<br>• Promoted a participatory approach as well as autonomy, enthusiasm & conscientiousness through continuous clinical and organizational audits, lectures, workshops & occasional meetings in person or online where nurses and other healthcare professionals could discuss potential adjustments or report on critical situations to improve patients' care<br>• Unit-level interventions, including training & enhancement of skills and psychological support with the establishment of a psychological help desk for staff, available every day on-site and remotely<br>• Surveillance of nurses and staff exposed to SARS-CoV-2 with nasopharyngeal swabs and successive serological samples<br>Implementation/usage:<br>• Initiatives were implemented starting March 2, 2020. Details are lacking on the number and nature of		7) Change (how organizational change is managed & communicated at work)		• Peer Support T0: 2.12 (.67) T1: 1.93 (.69) $t = 3.31$ $p = 0.001$<br>• Relationships T0: 2.23 (.88) T1: 2.04 (.68) $t = 3.42$ $p = 0.001$<br>• Role clarity T0: 1.71 (.52) T1: 1.69 (.60) $t = 0.26$ $p = 0.798$<br>• Change T0: 2.98 (.49) T1: 2.46 (.79) $t = 8.46$ $p<0.001$<br>Paired sample t-tests; lower scores indicate improvement. Results do not consider the level of adherence to the intervention, nor other potential confounders like age & having children. In Table 3, independent sample t-tests were used for comparisons of the psychosocial work environment according to sex & having	

Study (country)	Design	Participants analyzed	Participation rate at recruitment (%)	Loss to follow-up rate (%)	Intervention content & implementation	Intervention duration & timing of follow-up	Work exposures measured (physical, biological &/or psychosocial)	Mental health & related outcomes measured	Effect of intervention on work exposures	Effect of intervention on mental health & related outcomes
					changes, how they were applied in different areas of the hospital and on attendance and compliance by staff (e.g. number of training sessions, number who attended meetings, number & type of PPE, etc.)				children at each time point separately. Repeated-measures ANOVA was used to compare the psychosocial work environment according to marital status & work shift. Table 3 superscripts are not explained - authors were contacted on October 4, 2021 to clarify Table 3 results, but no reply.	
Zhu et al., 2020 (China)	Cross-sectional, no control group, but reference categories are used in regression analyses, i.e. workers unexposed to or unsatisfied with preventive measures	5,062 nurses (68%), doctors (20%) & clinical technicians (13%) of the largest tertiary hospital in Wuhan (Tongji Hospital) designated for the treatment of severe COVID patients (85% F)	80% (5,281/6,568)	N/A	Intervention content:<br>• Recognition measures (which authors call "hospital-based & department-based care"):<br>o Additional allowances for frontline staff<br>o Policy regarding promotions to the title "frontline worker"<br>o Verbal recognition (greetings) & support (reassurance) by hospital executives, nursing leaders & department chairs<br>o Acknowledging infections as work injuries<br>• IPC measures (which authors call "full coverage of all	Duration:<br>• Not specified, ~2 weeks is assumed: the shuttle service was meant to address the suspension of public transport by authorities on Jan 23, 2020 and the study was conducted 2 weeks after this date<br><br>Follow-up:<br>• Feb 8-10, 2020	Biological exposure<br>• Self-reported history of SARS-CoV-2 exposure (Do you feel that you have a history of exposure to SARS-CoV-2?)<br><br>• Confirmed or suspected COVID-19 infection (no details on the measure)	• Anxiety GAD-7≥8<br><br>• Acute stress in the past 7 days caused by a traumatic event, COVID-19 being the specific event IES-R>33<br><br>• Depression PHQ-9≥10	ND	**Associations between preventive measures & anxiety (GAD-7≥8), backwards stepwise elimination multivariate logistic regression analysis, men & women**<br>• Recognition measures AOR (95% CI): 0.76 (0.60 – 0.97); p = 0.03<br>• Satisfaction with IPC measures (vs. unsatisfied) AOR: 0.65 (0.50 – 0.85); p = 0.002<br>• Satisfaction with accommodation/food/shuttle service AOR: 0.69 (0.50 – 0.96); p = 0.03<br>• Effect of satisfaction with work shift arrangement not reported because NS in univariate analysis (p≥0.05)<br>Analysis adjusted for<br>o Gender (F vs. M)

Study (country)	Design	Participants analyzed	Participation rate at recruitment (%)	Loss to follow-up rate (%)	Intervention content & implementation	Intervention duration & timing of follow-up	Work exposures measured (physical, biological &/or psychosocial)	Mental health & related outcomes measured	Effect of intervention on work exposures	Effect of intervention on mental health & related outcomes
					departments for avoiding nosocomial infection”):<br>o Use of PPE in all departments regardless of presence of infected patients (medical protective clothing, N95 masks, goggles or disposable full-face shield, medical latex gloves, boot covers, medical working cap & gown)<br>o Disinfection of workplace<br>o Shutdown of central air conditioning<br>o Dissemination of facts about COVID-19 prevention<br>• Work shift arrangements<br>• Logistic support (shuttle, meals/hydration while working) & accommodation (hotels, hospital dormitories to reduce fear of infecting family members) arranged by hospital administrators when public transport suspended by authorities<br>• Virtual support group led by hospital psychiatry team (WeChat Balint group)<br>Implementation/usage:<br>• No details on how preventive measures were applied and used by workers with respect					o Years worked (2-5, 6-10, >10 years worked vs. <2 years)<br>o Living with family members<br>o Past medical history (non-communicable chronic diseases, history of mental disorders vs. in good health)<br>o Meets WHO recommendation for physical activity of ≥60 min./day moderate to high intensity, 3 times/week (yes vs. no)<br>o Family/relatives confirmed/suspected of being infected<br>o Self-reported history of SARS-CoV-2 exposure (agree/unsure vs. disagree)<br>o Thought of resigning because of COVID-19 outbreak (agree/unsure vs. disagree)<br>o Worried for your life if infected (agree/unsure vs. disagree)<br>o Worried about myself or family being infected<br>o Feeling that relatives have avoided contact with you because of your work (avoided/unsure vs. didn’t avoid)<br>**Associations between preventive measures & acute stress (IES-R>33), backwards stepwise elimination multivariate logistic regression analysis, men & women**<br>• Recognition measures AOR (95% CI): 0.76 (0.60 – 0.97); $p = 0.024$<br>• Satisfaction with IPC measures AOR: 0.69 (0.53 – 0.89); $p = 0.004$

Study (country)	Design	Participants analyzed	Participation rate at recruitment (%)	Loss to follow-up rate (%)	Intervention content & implementation	Intervention duration & timing of follow-up	Work exposures measured (physical, biological &/or psychosocial)	Mental health & related outcomes measured	Effect of intervention on work exposures	Effect of intervention on mental health & related outcomes
					to work shift arrangements, IPC measures & accommodation/logistic support (food/shuttle service)<br>• 91% of respondents reported having received allowances, verbal recognition & support from their supervisors<br>• ≥80% were satisfied with IPC measures, work shift arrangements and accommodation/logistic support<br>• Only 5% joined the virtual support groups					• Effect of satisfaction with accommodation/food/shuttle service not reported because NS in univariate analysis<br>• Satisfaction with work shift arrangement AOR: 0.45 (0.33 – 0.63); p<0.001<br>• Uncertain if satisfied with work shift arrangement AOR: 0.65 (0.46 – 0.92); p = 0.02<br>Analysis adjusted for<br>o Gender (F vs. M)<br>o Master's degree or higher<br>o Nurse or technician (vs. doctor)<br>o Years worked (2-5, 6-10, >10 years worked vs. <2 years)<br>o Working in isolation ward or being off work/in isolation (vs. working in non-isolation ward)<br>o Past medical history (non-communicable chronic diseases, history of mental disorders vs. in good health)<br>o Having children (1 child, ≥2 children vs. no children)<br>o Family/relatives confirmed/suspected of being infected<br>o Self-reported history of SARS-CoV-2 exposure (agree/unsure vs. disagree)<br>o Thought of resigning because of COVID-19 outbreak<br>o Worried for your life if infected (agree/unsure vs. disagree)<br>o Feeling that relatives have avoided contact with you because of your work (avoided/unsure vs. didn't avoid)

Study (country)	Design	Participants analyzed	Participation rate at recruitment (%)	Loss to follow-up rate (%)	Intervention content & implementation	Intervention duration & timing of follow-up	Work exposures measured (physical, biological &/or psychosocial)	Mental health & related outcomes measured	Effect of intervention on work exposures	Effect of intervention on mental health & related outcomes
										**Associations between preventive measures & depression (PHQ-9≥10), backwards stepwise elimination multivariate logistic regression analysis, men & women**<br>• Recognition measures AOR (95% CI): 0.69 (0.52 – 0.90); p = 0.007<br>• Satisfaction with IPC measures AOR: 0.70 (0.51 – 0.95); p = 0.02<br>• Satisfaction with accommodation/food/shuttle service AOR: 0.67 (0.47 – 0.97); p = 0.03<br>• Satisfaction with work shift arrangement AOR 0.48 (0.34 – 0.67); p<0.001<br>• Uncertain if satisfied with work shift arrangement AOR: 0.61 (0.42 – 0.89); p = 0.01<br>Analysis adjusted for<br>o Gender (F vs. M)<br>o Years worked (2-5, 6-10, >10 years worked vs. <2 years)<br>o Past medical history (non-communicable chronic diseases, history of mental disorders vs. in good health)<br>o Drinking history (yes vs. no)<br>o Family/relatives confirmed/suspected of being infected<br>o Suspected/confirmed COVID-19 infection<br>o Self-reported history of SARS-CoV-2 exposure (agree/unsure vs. disagree)<br>o Thought of resigning because of COVID-19 outbreak

Study (country)	Design	Participants analyzed	Participation rate at recruitment (%)	Loss to follow-up rate (%)	Intervention content & implementation	Intervention duration & timing of follow-up	Work exposures measured (physical, biological &/or psychosocial)	Mental health & related outcomes measured	Effect of intervention on work exposures	Effect of intervention on mental health & related outcomes
										o Worried for your life if infected (agree/unsure vs. disagree)<br>o Feeling that relatives have avoided contact with you because of your work (avoided/unsure vs. didn't avoid)<br>**Gender-stratified regression analyses**<br>• In men (n=758), only satisfaction with IPC measures (and being uncertain if satisfied) was retained in models and was associated with reduced likelihood of anxiety [AOR 0.42 (0.23 – 0.75)] & acute stress [AOR 0.39 (0.21 – 0.72)], but an unexplained increase in likelihood of depression [AOR 2.52 (1.18 – 5.36].<br>• In women (n=4,304):<br>o Recognition measures were associated with reduced likelihood of anxiety [AOR 0.55 (0.33 – 0.93)], acute stress [AOR 0.70 (0.54 – 0.91)] & depression [AOR 0.68 (0.50 – 0.92)<br>o Satisfaction with work shift arrangements was associated with reduced likelihood of acute stress [AOR 0.34 (0.24 – 0.49) & depression [AOR 0.40 (0.28 – 0.59). Being uncertain if satisfied was also associated with reduced likelihood of acute stress [AOR 0.53 (0.36 – 0.78) & depression [AOR 0.56 (0.38 – 0.84).<br>o Satisfaction with accommodation/food/shuttle service was associated with reduced likelihood of

Study (country)	Design	Participants analyzed	Participation rate at recruitment (%)	Loss to follow-up rate (%)	Intervention content & implementation	Intervention duration & timing of follow-up	Work exposures measured (physical, biological &/or psychosocial)	Mental health & related outcomes measured	Effect of intervention on work exposures	Effect of intervention on mental health & related outcomes
										depression [AOR 0.55 (0.38 – 0.81).

AOR: adjusted odds ratio; C: control; CDC: Centers for Disease Control and Prevention; CI: confidence interval; F: female; GAD: Generalized Anxiety Disorder; HADS: Hospital Anxiety and Depression Scale; HSE-MSIT: Health & Safety Executive Management Standards Indicator Tool; I: intervention; ICU: intensive care unit; IES-R: Impact of Event Scale-Revised; IPC: Infection prevention and control; K10: Kessler 10-item psychological distress scale; MBI-EE: Maslach Burnout Inventory - Emotional Exhaustion subscale; N/A: not applicable; ND: not documented; NS: not significant; NQoL-SAT-P: Nurses Quality of Life Scale - Satisfaction Profile; PHQ-9: Patient Health Questionnaire; PPE: personal protective equipment; PSQI: Pittsburgh Sleep Quality Index; SARS: Severe Acute Respiratory Syndrome; SD: standard deviation; UWES-9: Utrecht Work Engagement scale; WEMWBS: Warwick-Edinburgh Mental Wellbeing Scale; WHO: World Health Organization
